# Fotemustine: A Third-Generation Nitrosourea for the Treatment of Recurrent Malignant Gliomas

**DOI:** 10.3390/cancers4010077

**Published:** 2012-02-01

**Authors:** Patrick Beauchesne

**Affiliations:** Neuro-oncology/Neurology, University Hospital of Nancy, Hôpital CENTRAL, CO N 34,54035 Nancy cedex, France; E-Mail: beauchesneP@wanadoo.fr; p.beauchesne@chu-nancy.fr; Tel.: +33-3-8385-1688; Fax: +33-3-8385-2734

**Keywords:** malignant gliomas, recurrent tumors, chemotherapy, fotemustine, salvage therapy, second-line treatment

## Abstract

Malignant gliomas account for approximately 60% of all primary brain tumors in adults. The prognosis for patients with malignant glioma has not changed significantly in recent years. Despite debulking surgery, radiotherapy and cytotoxic chemotherapy, the median survival time is nine to 12 months, and very few, if any, patients are cured from this illness. Fotemustine is an alkylating agent characterized by the grafting of a phosphonoalanine group onto the nitrosourea radical with consequent high lipophilicity and improved diffusion through the cell membrane and the blood-brain barrier. Fotemustine has been registered for use in two indications: disseminated malignant melanoma, including cerebral metastases, and primary brain tumors. Fotemustine is currently used in Europe, particularly in France and Italy, as a salvage therapy for recurrent malignant gliomas. Myelosuppression, leucopenia and thrombocytopenia are the most frequent side effects of treatment with fotemustine. The objective response to this treatment is between 26% and 70%, and the reported median survival time is 10 months. New drug combinations containing fotemustine and angiogenesis inhibitors, such as bevacizumab, are currently under development. In this review, we describe all the combinations of fotemustine currently used in clinical practice for recurrent malignant gliomas.

## 1. Introduction

Malignant gliomas (high-grade gliomas) are the most common brain tumors in adults, accounting for approximately 60% of them [1,2,3,4]. Based on the neuropathological classification of the WHO, there are three distinct histological types: anaplastic astrocytoma (AA), anaplastic oligodendroglioma (AO) and glioblastoma multiforme (GBM) [1,2,3,4]. Malignant gliomas, especially GBM, are among the most difficult cancers to treat, with a short survival time and a poor response to chemotherapeutic drugs [1,2,3,4]. For AA and GBM, the standard of care for many decades has consisted of surgical resection of as much of the tumor as considered to be safe, followed by radio- and chemo-therapy [1,2,3,4]. A new standard treatment strategy for GBM has recently been defined by the EORTC Phase III trial, in which two groups of randomized patients receive either temozolomide (TMZ) concomitant and adjuvant to cerebral radiotherapy, or radiotherapy alone [5]. A significant increase in overall survival was observed in the radiotherapy plus TMZ group as compared to the radiotherapy alone group. Median survival times were 14.6 and 12.1 months, respectively [5].

For AO, the options of the therapies are based on the 1p19q deletion status; for 1p19q deletions, cranial radiotherapy plus chemotherapy or chemotherapy alone should be recommend, no significantly difference in overall survival was found [6]. In cases with no deletion, cerebral radiotherapy plus chemotherapy is the optional therapy [6].

The median survival time for GBM patients is from nine to 12 months, and the median survival times for AA and AO are from 24 to 36 months, and 60 months, respectively [1,2,3,4,5,6].

Despite the aggressive standard treatment, the rate of recurrence of malignant gliomas is very high. There are various reasons for this poor prognosis and therapeutic resistance:

The blood-brain barrier delimits a pharmacological sanctuary;
Glioma tumor cells and capillaries express multidrug resistance proteins;
Glioma cells present genetic, molecular and metabolic heterogeneity;
There is chemo-resistance to commonly administered alkylating drugs;
There is a cellular immunity defect;
The presence of a tumor’s stem cells within malignant gliomas is often responsible for the failure of conventional therapies [7].

Moreover, initial radiation therapy can trigger several events, such as direct DNA damage, which could lead either to cell death or to mutations in survivor cells, which in turn might result in the apparition of resistant clones and lead to recurrence [7].

Presently, there is no consensus therapy recommended for the treatment of recurrent malignant gliomas, as well as no optimal sequence of therapies has been defined yet [8]. A second debulking surgery could be proposed as well as a second course of radiotherapy if a local treatment should be applicable [8]. These satisfactory therapeutic options depend on the site and pattern of the relapse, as well as on the time elapsed since the prior therapy.

Different schedules of re-irradiation could be proposed; stereotactic radiation therapy (SRS), hypofractionated stereotactic radiotherapy (FSRT), or a combination of chemotherapy and hypofractionated radiotherapy [8]. Frequently, the local treatment is not possible, so, the main treatment for recurrent malignant gliomas is systemic chemotherapy. Different regimens containing various anti-neaplastic agents such as procarbazine, platinum, etoposide, temozolomide or various combinations of those, were proposed and used [8]. The reported response rate varies between 20% and 30%, and the median survival time is approximately six months [8,9]. Recent Phase II clinical trials have demonstrated the beneficial activity of nitrosourea agents such as fotemustine in recurrent malignant gliomas [9]. In this review, we describe the different modes of administration of fotemustine and the different combinations of fotemustine used in clinical practice.

## 2. Fotemustine

**Figure 1:**
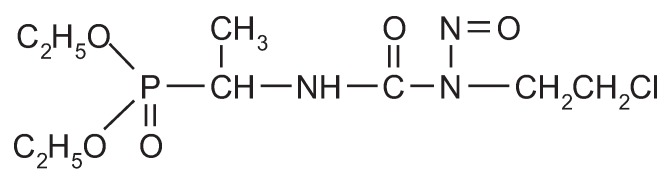
Structural formula of fotemustine.

Fotemustine (diethyl 1-{1-[3-(2-chloroethyl)-3-nitrosoureido]} ethylphosphonate RS; molecular formula: C_9_H_19_ClN_3_O_5_P; molecular weight: 315.7; Figure 1) is a novel chloroethylnitrosourea characterized by a high liposolubility and a low molecular weight, which favor its passage across the blood-brain barrier. Its chemical structure includes an alanine phosphonic ester [10]. The antitumor activity of fotemustine is related to its ability to alkylate DNA. Its *in vitro* or *in vivo* pharmacological activity is similar or greater than that of other nitrosoureas [10,11,12,13]. Significant activity has been found in mice xenograft models of human primary cerebral tumors after fotemustine intraperitoneal administration [14]. Fotemustine rapidly crosses the blood-brain barrier, and its plasma pharmacokinetic pattern in animals and humans is linear and biphasic, with a terminal half-life of 29 minutes in humans. Metabolism occurs mainly through rapid non-enzymatic chemical decomposition into the carbonium ion, which is responsible for the alkylating activity, and into the isocyanate group, which has carbamoylating properties, and results in the formation of chloroethanol [10]. Fotemustine is highly photosensitive, and it is thus essential to protect the solution from light while preparing it and administering it to patients [10]. Fotemustine is a cytotoxic alkylating agent of the lipophilic chloroethylnitrosourea group [10].

## 3. Pre-Concurrent Radiotherapy-Temozolomide Era

The first study that tested fotemustine in recurrent malignant gliomas was reported by Frenay *et al.* [15]. It included 38 patients of a heterogeneous histology, and the tumor distribution was: 21 GBM, nine AA, six degenerated low-grade gliomas, one pinealoblastoma and one patient with no histological data [15]. The administration schedule was standard, and consisted of the administration of one dose of 100 mg/m^2^ of fotemustine per week for three consecutive weeks (induction treatment), followed by a five-week rest period. After this, one dose of fotemustine was administered every three weeks until tumor progression, emergence or apparition of side effects (maintenance treatment) [15]. All patients received the induction treatment, but only 13 received the maintenance treatment (cycles ranging from one to 13). The most common side effect was a reversible, cumulative and delayed hematological toxicity. Grade 3/4 thrombocytopenia and leukopenia were observed in 23 and 17% of patients, respectively [15]. Prior chemotherapy was associated with a 30% increased rate of both thrombocytopenia and leukopenia. A partial objective response was reported in 9 patients; three with GBM, three with AA, and three with a degenerated low-grade glioma [15]. Three patients had previously been treated with chemotherapy, including nitrosourea. The median survival time for these responder patients was 40 weeks after their inclusion, and the median response duration was 32.7 weeks [15]. Stabilization was observed in 18 patients (11 GBM, five AA, and two low-grade gliomas), and the median overall survival time was 42.5 weeks; the median duration of stabilization was 21 weeks, and four patients were still alive one year after their inclusion in the study [15]. Ten patients did not respond to the treatment, and their median survival time was only 15.2 weeks [15]. It was postulated that fotemustine might be used for the treatment of recurrent malignant gliomas (Table 1).

**Table 1:** Fotemustine before temozolomide era.

Authors	Number of Patients	Schedule	Toxicity Grade 3–4 Thrombocytopenia and Leucopenia	Response Rate
Frenay *et al*. [15]	38	Standard	23 and 17% of patients	23%
Khayat *et al*. [16]	8	Intra-arterial	14% of patients	12.5%
Malhaire *et al*. [17]	22	Standard	2 and 4 patients	50%
Mousseau *et al*. [18]	34	Standard	NA	70%
Boiardi *et al*. [19]	16	Escalating doses and procarbazine	1 and 1 patients	50%
Fazen-Döner *et al*. [20]	31	Standard and dacarbazine	1 and 3 patients	54.8%

Khayat *et al.* tested intra-arterial infusion of fotemustine in eight patients with recurrent malignant gliomas, as intra-arterial delivery of anti-cancer drugs could be a rational approach for localized diseases such as primary brain tumors [16]. Fotemustine was diluted in 5% glucose, with no alcohol as a solvent in order to improve ocular tolerance. The treatment consisted of one intra-arterial infusion every six weeks [16]. Only one partial response was reported. The hematological toxicity was mild; leukopenia or thrombocytopenia were only observed in 14% of the patients [16]. The main toxicity issue reported was vision impairment (blindness, loss of vision) (Table 1) [16].

Malhaire *et al.* tested fotemustine in 22 recurrent malignant glioma patients, including 19 GBM and three AA. No prior chemotherapy was administered, and all patients responded favorably to postoperative radiotherapy [17]. The fotemustine schedule was the same as previously described. An objective response was observed in 11 patients, including four partial responses and seven stabilizations [17]. Grade 3/4 leukopenia and thrombocytopenia were observed in two and four patients, respectively. Only one patient discontinued chemotherapy after five cycles of fotemustine (hematological toxicity—grade 3 thrombocytopenia) [17]. The median survival time was 9.4 months for responder patients, and the time between treatment and response or stabilization was 6.5 months [17]. The median survival time for the patients who failed to respond to fotemustine was only five months. Again, these preliminary results were encouraging (Table 1).

Another study from France tested fotemustine on tumor recurrence in 34 chemotherapy-naive patients with malignant gliomas [18]. The rate of objective response, including partial responses and stabilizations, was promising, as it was observed in 24 patients (70%) [18]. The median overall survival time was 40 weeks, similar to previous studies (Table 1).

Boiardi *et al.*, in a Phase I clinical trial that evaluated the maximum tolerated dose (MTD) and toxicity profile of fotemustine with a fixed dose of procarbazine (PCZ), studied 16 recurrent malignant gliomas [19]. Four dosage levels of fotemustine were tested: 50, 75, 100 and 125 mg/m^2^. All patients had received prior radiation therapy after a neurosurgical procedure (all but one underwent a tumor resection), and 15 patients had previously received at least one previous chemotherapy (nitrosourea) [19]. The median time between the last chemotherapy and the beginning of the new chemotherapy (fotemustine-PCZ) was 4.8 months. The efficacy of fotemustine was studied, and one partial response and seven stabilizations were reported (50%). The estimated median overall survival time was 9.7 months, and the one-year survival rate was 43.75% (Table 1) [19].

Fazeny-Dörner *et al.* tested the combination of dacarbazine (D) and fotemustine in 31 recurrent GBM patients [20]. All patients had received prior radiation therapy and first-line chemotherapy (nitrosourea). Progressive or recurrent tumors were diagnosed through computed tomography scan (CT) or magnetic resonance imaging (MRI), and dacarbazine and fotemustine were administered at 200 mg/m^2^ and 100 mg/m^2^, respectively [20]. One partial response and 16 stabilizations were reported (54.8%), and the median overall survival time (calculated from the onset of the D/fotemustine treatment) was 45 weeks [20]. Grade 3/4 leukopenia and thrombocytopenia were observed in one and three patients, respectively, and grade 2 thrombocytopenia was noted in three patients [20]. This D/fotemustine combination proved to be effective even in patients previously treated with nitrosourea (Table 1).

## 4. Post-Concurrent Radiotherapy-Temozolomide Era

Despite the new gold-standard treatment for GBM, *i.e*., temozolomide concomitant and adjuvant to cerebral radiotherapy, nearly all patients treated for GBM experienced tumor recurrence. There is no established chemotherapy strategy for the treatment of recurrent malignant gliomas. The treatment recommendations after temozolomide chemotherapy are based exclusively on non-controlled Phase II studies. Scoccianti *et al.* tested fotemustine as salvage chemotherapy in 27 patients with recurrent GBM, all treated by cerebral radiotherapy with concomitant and adjuvant TMZ (for six cycles) [21]. At the progression or recurrence of the tumor (proved by cranial MRI), all patients underwent chemotherapy with fotemustine administered intravenously at 100 mg/m^2^ every week for three consecutive weeks (induction phase), and then every three weeks (maintenance phase). Eight partial responses and five stabilizations were observed. The rate of objective response was 48.1%. The toxicity was as expected and mainly hematological (three cases of grade 3 thrombocytopenia, and one case of grade 4 leukopenia) [21]. The median survival time after fotemustine treatment onset was 9.1 months, and the median survival time after the initial diagnosis was 21.1 months. Progression-free survival rate at six months was 48.15%, and the median time to progression was 5.7 months [21]. Thus, fotemustine showed a therapeutic effect in the treatment of recurrent patients pretreated with TMZ.

The Italian cooperative neuro-oncology group conducted a Phase II study testing fotemustine in patients with progressive GBM, after radiotherapy with concomitant and adjuvant TMZ chemotherapy. Forty-three patients were included in the trial (29 males, 14 females), and they received fotemustine at the classical regimen (induction and maintenance phases) [22]. An MGMT promoter methylation analysis was performed on tumor tissues sampled from the initial neuro-surgery before radiotherapy and TMZ. The median time between the end of TMZ chemotherapy and the start of fotemustine treatment was 1.5 months (ranging from one to 43 months). Hematological toxicity was observed. After the induction phase, grade 3/4 thrombocytopenia and leukopenia were noted in 20.9% and 16% patients, respectively, and grade 3/4 lymphopenia was noted in four patients [22]. The dosage of fotemustine was then reduced to 75 to 100 mg/m^2^/day, and the rate of grade 3/4 thrombocytopenia after this amendment fell to 15% [22]. Twenty-one patients started the maintenance phase and received a median of two cycles (ranging from one to 14). No grade 3/4 thrombocytopenia was observed, and grade 3/4 leukopenia was noted in five patients (23.8%) [22]. Among the assessed patients, three partial responses and 15 stabilizations were reported (42%). The overall survival time was six months, and no difference was found between patients with methylated and unmethylated MGMT promoters. The survival time of patients who started fotemustine at least three months after TMZ completion was longer than those who started immediately after TMZ administration (8.4 *vs.* 5.4 months) [22]. The progression-free survival rate at six months was 20.9%, and the median time to progression was 1.7 months, the progression-free survival of patients who started fotemustine at least 3 months after TMZ completion was significantly higher than that of those who initiated fotemustine immediately after (30.7% *vs.* 16.7%) [22]. This trial represents a new benchmark for nitrosourea activity (Table 2).

**Table 2:** Fotemustine post-temozolomide era.

Authors	Number of Patients	Schedule	Toxicity	Response Rate
Brandes *et al*. [22]	43	Standard	20.9 and 16% of patients	42%
Fabrini *et al*. [23]	50	Standard	4 and 1 patients	62%
Silvani *et al*. [24]	54	Combination of fotemustine and procarbazine	4 and 2 patients	
Fabi *et al*. [26]	40	65 to 100 mg/m^2^ of fotemustine	8 and 6 patients	47.5%
Fabi *et al*. [26]	40	60 mg/m^2^ of fotemustine, every 3 weeks	3 and 4 patients	52.5%
Addeo *et al*. [9]	40	80 mg/m^2^ of fotemustine, every 2 weeks	7 and 3.5% of patients	65%

Fabrini *et al.* treated 50 patients suffering from recurrent malignant gliomas (31 males and 19 females who had received the standard therapy) with fotemustine through an induction phase (on days 1-8-15) and maintenance phase (one infusion every three weeks after a 4–6 week rest period) [23]. All but 9 patients were GBM (WHO grade III astrocytoma) and received concomitant radiochemotherapy and adjuvant chemotherapy with TMZ. Grade 3 astrocytoma patients received radiotherapy following by TMZ chemotherapy. The median time span between diagnosis and fotemustine induction was 12.5 months (ranging from 6.5 to 79.4 months). Only 29 patients entered the maintenance phase of fotemustine, and hematological toxicity was reported, as in the cases reported above. Grade 3 thrombocytopenia was observed in four patients (8%), grade 4 leukopenia in one patient (2%), and grade 3 lymphopenia in one patient (2%) [23]. One patient suffered an intralesional hemorrhage. One complete response, eight partial responses and 22 stabilizations were reported after the induction phase of fotemustine (the rate of objective response was 62%) [23]. The median overall survival time after fotemustine treatment was approximately 8.1 months, and the median overall survival time after diagnosis was 24.5 months. Survival at one year from initial diagnosis was 80.7% [23]. The progression-free survival rate at six months was 51.5%, and the median time to progression was 6.1 months. Again, a significant effect of fotemustine for the treatment of recurrent GBM patients was reported (Table 2).

Fotemustine combined with procarbazine was proposed as a salvage therapy in the treatment of recurrent GBM patients pretreated with TMZ. Silvani *et al.* conducted this Phase II study in 54 recurrent GBM patients (41 males and 13 females). Procarbazine was orally administered at a dosage of 450 mg on days 1 and 2, and 300 mg on day 3. Fotemustine was given on day 3, three hours after the last procarbazine intake, at a dose of 110 mg/m^2^. This treatment was repeated every five weeks [24]. Six partial responses and 29 stabilizations were reported (64.9%). The mean amount of cycles administered to patients was 2.38 (ranging from one to seven cycles). Grade 4 thrombocytopenia and neutropenia were reported in four and two of all cycles, respectively. One patient experienced lower limb deep vein thrombosis, another one developed a pneumopathy, and grade 3 liver toxicity was observed in two patients [24]. The median overall survival time after fotemustine-procarbazine treatment onset was 28.7 weeks (7.1 months), and the median survival time after the initial diagnosis was 20.8 months. Progression-free survival at six months was 26.7%, and the median time to progression was 19 weeks (4.7 months) (Table 2) [24].

In order to address the importance of fotemustine dosage in the treatment of recurrent malignant gliomas, Fabi *et al.* carried out a Phase II clinical study to test the activity of different doses of fotemustine in patients with recurrent malignant gliomas [25]. Forty patients were included; the histology was 14 GBM, 11 AA, seven anaplastic oligoastrocytomas, and eight AO. Fotemustine was the second- or third-line chemotherapy in these patients [25]. Fotemustine was administered intravenously at doses ranging from 65 to 100 mg/m^2^ weekly for three consecutive cycles (induction phase), followed by a five-week rest period. Fotemustine intake was then resumed with tri-weekly cycles at doses ranging from 75 to 100 mg/m^2^ [25]. MGMT promoter methylation analysis was performed on tumor tissue taken from the initial neurosurgery process. Nineteen tumor tissues were analyzed. The median number of fotemustine cycles received was six, ranging from four to eight. Eight partial responses and 11 stabilizations were reported; the rate of objective response was 47.5% [25]. The median overall survival time after fotemustine treatment was estimated to be about 30 months. Overall survival was significantly higher among patients who responded to fotemustine than among patients who failed to respond. Progression-free survival at six months was 27%, and the median time to progression was four months [25]. MGMT promoter was methylated in 12 cases with three partial responses and nine stabilizations. The doses of fotemustine started at 65 to 75 mg/m^2^ (induction phase) and increased to 75 to 85 mg/m^2^ (maintenance phase) for an activity comparable to that of the standard schedule (Table 2).

In a second trial, Fabi *et al.* tested lower doses of fotemustine. The induction phase consisted in the weekly administration of fotemustine to patients with recurrent malignant gliomas for three consecutive weeks at 60 mg/m^2^, followed by a maintenance phase at 75 mg/m^2^ every three weeks, after a rest period [26]. Forty patients pretreated with fewer than two chemotherapy strategies were recruited. The histology was 30 GBM, six AA and four AO. Eight partial responses and 13 stabilizations were reported, for a disease control rate of 52.5% [26]. Hematological toxicity and grade 3 thrombocytopenia (n = 3) and leukopenia (n = 4) were reported in some patients. The median PFS was three months, and the PFS at six months was 21%. The median overall survival time was six months, and the survival rate at 12 months (after the start of fotemustine treatment) was 31.2% [26]. This study showed that low-dose treatments of fotemustine for recurrent malignant glioma patients have an activity comparable to that of the full-dose regimen, but with a significantly lower myelotoxicity (Table 2).

Addeo *et al.* reported a very interesting study in which fotemustine was administered at a dose of 80 mg/m^2 ^every two weeks for five consecutive weeks (induction phase) to recurrent malignant glioma patients, followed by a four-week rest period, and a maintenance phase consisting of fotemustine at 80 mg/m^2^ every four weeks [9]. Forty patients (29 males and 11 females) were included, and all patients had a histologically proven recurrent GBM. All patients had previously received treatments with TMZ concomitant and adjuvant to cerebral radiotherapy, and the median time between the initial diagnosis and the fotemustine induction phase was 11.6 months [9]. As expected, there was hematological toxicity. Grade 3 thrombocytopenia and leukopenia were reported during the induction phase in three and one patients respectively, and during the maintenance phase, grade 3 thrombocytopenia and leukopenia were 7 and 3.5%, respectively [9]. No interruption of fotemustine due to toxicity was observed in this study. Among the assessable patients, one complete response, nine partial responses, and 16 stabilizations were reported, for a disease control rate of 65% [9]. The median progression-free survival was 6.7 months, and progression-free survival at six months was 39%. The median overall survival time after fotemustine treatment onset was 11 months [9]. The protracted low doses of fotemustine showed significant benefit in the treatment of recurrent GBM patients without major toxicity, opening the door to a new period of clinical studies testing the combination of low doses of fotemustine and targeted therapy agents (Table 2).

## 5. Combination of Fotemustine and Targeted Therapy Agents

Bevacizumab (Avastin^®^), a humanized monoclonal antibody that binds to vascular endothelial growth factor (VEGF) and inhibits its activity, has demonstrated a cytotoxic activity in various cancers, such as colorectal, lung, renal and breast cancer [27]. GBM, as well as the previously listed tumor types, overexpress VEGF, and preclinical studies have shown that bevacizumab is effective for the treatment of recurrent GBM [27]. Moreover, bevacizumab has been shown to provide a clinical benefit for GBM patients. Bevacizumab received approval from the FDA, but not the EMA, for the treatment of progressive GBM patients as single-agent following prior therapy.

Soffietti *et al.* performed a Phase II study testing the treatment of recurrent malignant glioma patients with a combination of fotemustine and bevacizumab [28]. The patients included in the study had a recurrent GBM and had previously benefited from surgery, radiotherapy and concomitant and adjuvant chemotherapy with TMZ. Bevacizumab was administered intravenously. The treatment consisted of an induction phase with bevacizumab at a dose of 10 mg/kg on days 1 and 15, and fotemustine at a dose of 75 mg/m^2^ on days 1 and 8, followed by a maintenance phase with administration of bevacizumab at a dose of 10 mg/kg and fotemustine at a dose of 75 mg/m^2^ every three weeks, after a three-week rest interval [28]. MRI assessment was performed one month after starting chemotherapy, and once every two months thereafter. Fifty-four patients were enrolled (35 males and 19 females, median age 57). Two complete responses, 24 partial responses and 22 stabilizations were reported; the disease control rate was 89% [28]. Also, an improvement of the neurological status was noted. 22% of patients experienced grade 3/4 thrombocytopenia and leukopenia, for which fotemustine was discontinued. One stroke, one intratumoral hemorrhage, one gastrointestinal perforation and one pulmonary embolism were observed and associated with bevacizumab treatment, and in these cases, bevacizumab was stopped [28]. The median progression-free survival was 5.29 months, and progression-free survival at six months was 44%. The median overall survival time after the start of fotemustine treatment was 9.13 months. 31% of patients survived longer than 12 months [28]. This combination of bevacizumab and fotemustine proved to be safe and promising, and warrants further clinical studies to confirm these preliminary results.

## 6. Conclusions

In conclusion, fotemustine, an old nitrosourea drug, shows undeniable efficacy in the treatment of pretreated recurrent malignant gliomas. The overall rate of objective response was approximately 45%, with an acceptable hematological toxicity. In an effort to reduce toxicity, a new schedule was developed by Addeo *et al.* with fotemustine administration at 80 mg/m^2 ^every two weeks for five consecutive weeks (induction phase), followed by a four-week rest period, and a maintenance phase at 80 mg/m^2^ every four weeks. The combination of fotemustine and a targeted agent such as bevacizumab seems promising and warrants further studies. Fotemustine could be beneficial in the treatment of recurrent malignant gliomas.
